# Plasma Cell Leukemia: Definition, Presentation, and Treatment

**DOI:** 10.1007/s11912-019-0754-x

**Published:** 2019-01-28

**Authors:** Michael Tveden Gundesen, Thomas Lund, Hanne E. H. Moeller, Niels Abildgaard

**Affiliations:** 1 Department of Hematology, Odense University Hospital, Kloevervaenget 10, 12th floor, DK-5000 Odense C, Denmark; 2 Department of Pathology, Odense University Hospital, JP Winsløvs vej 15, 2th floor, DK-5000 Odense C, Denmark; 3 Department of Clinical Research, University of Southern Denmark, Odense, Denmark

**Keywords:** Plasma cell leukemia, Diagnosis, Molecular biology, Cytogenetics, Treatment, Prognosis

## Abstract

**Purpose of Review:**

We discuss current topics on the definition of plasma cell leukemia and the distinction between plasma cell leukemia and multiple myeloma. Moreover, we review the latest literature on how to treat plasma cell leukemia.

**Recent Findings:**

Plasma cell leukemia is clinically and genetically distinct from multiple myeloma. Plasma cell leukemia is defined by the observation in blood of more than 20% clonal plasma cells by differential count of the leucocytes or by counting more than 2 × 10^9^ per liter circulating clonal plasma cells. However, patients with lower levels of circulating plasma cells have the same adverse prognosis, which challenges the disease definition. Survival has improved after implementation of high-dose chemotherapy with stem-cell support, bortezomib, and lenalidomide in the treatment; yet, the prognosis remains poor. The results of allo-transplants have been disappointing.

**Summary:**

The diagnostic criteria of PCL are currently discussed in the international myeloma community. Despite some improvement in survival, the prognosis remains adverse. New, more targeted treatment modalities, including immunotherapies, will hopefully improve the outcome in the near future.

## Introduction

Plasma cell leukemia (PCL) is a rare and aggressive form of leukemia and plasma cell dyscrasia. PCL can be divided into primary PCL (PCL) and secondary PCL (sPCL) following previously diagnosed multiple myeloma (MM); the latter typically occurring at a late and advanced stage of MM. In this review, we will primarily focus on primary PCL, but also mention sPCL, when appropriate. PCL is uncommon, but with some differences in reported incidence in different populations. In the American SEER database between 1973 and 2009, PCL accounted for approximately 0.6% of the MM cases, which translates to about 1200 patients a year in the USA [1]. In the European HAEMACARE project, the crude incidence was found to be 0.4 per million, and PCL accounted for approximately 0.5% of the MM cases [2]. These numbers are lower than earlier reported estimates of 2–4% of MM patients [3–6]. The Danish National Multiple Myeloma Registry has registered all PCL cases since 2005 [7]. It covers the entire Danish population in a country with a free and public health care system, and data completeness is almost 100%. From 2005 to 2015, the crude Danish PCL incidence was 1.2 per million and accounted for approximately 2% of the MM cases [7]. Historically, primary PCL has been more common than sPCL. However, in recent years, the number of cases of sPCL has increased [6]. This is probably caused by the improved survival of MM; more patients live long enough for the clone to evolve into sPCL. A review from 2018 found that the prevalence of MM might have as much as tripled in recent years due to an aging population and improved survival [8]. Also, more MM patients receive several lines of treatment, which can potentially contribute to clonal selection and thereby co-drive the development of sPCL [9].

The prognosis of primary PCL has generally been dismal with reported median overall survival (OS) below 1 year [1]. With the use of novel treatments and autologous stem cell transplantation (ASCT), this has improved somewhat, although the prognosis remains poor. Novel treatment modalities, including immunotherapy and cellular therapy are under evaluation, and hopefully, these new technologies will show efficacy in PCL and improve the prognosis.

In this review, we will discuss some controversies in the definition of PCL and focus on clinical presentation, disease biology, and treatment.

## Definition and Diagnosis of Plasma Cell Leukemia

**Fig. 1:**
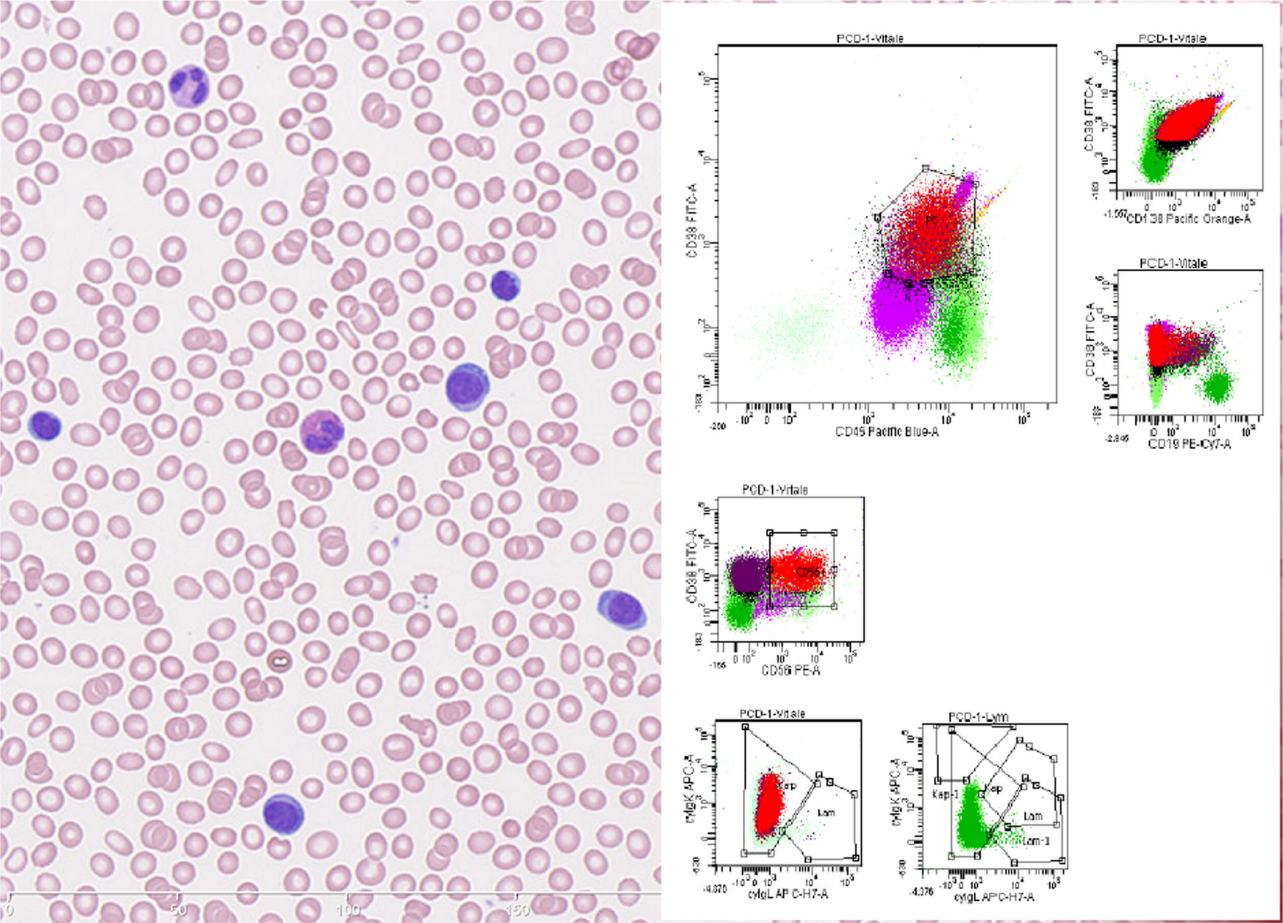
Left: Blood smear showing plasma cells constituting > 20% of total leukocytes. The plasma cells are atypical with high nuclear/cytoplasmic ratio. Right: flow cytometry histograms of blood. The neoplastic plasma cells indicated in red and purple (CD56 positive and negative fraction respective) express CD138, bright CD38, CD45, cytoplasmic kappa and are negative for CD19 and cytoplasmic lambda

The diagnostic definition of PCL has traditionally been based on Kyle’s criteria from 1974 [10]. In this seminal paper, PCL was defined by at least 20% circulating plasma cells and a total plasma cell count in peripheral blood of at least 2 × 10^9^/l, thereby identifying a leukemic subtype of MM with a particularly poor prognosis. Since, this paper has provided the framework for the diagnosis of PCL [10] However, the definition is still under debate. Some studies use only one of the original two requirements to define PCL, and several recent studies question whether a lower threshold of total plasma cells might better risk classify a subgroup of MM patients. Moreover, advances in flow cytometry allow better characterization and clonality assessment of plasma cell populations [1, 11–16]. The morphology and immunophenotype of the malignant PCs in PCL, MM, and sPCL are not distinguishable. The expression of the plasma cell markers CD138 and CD38 does not differ between the groups. Nevertheless, significant differences have been observed. The adhesion molecule CD56 is more frequently found to be positive in MM, and the B cell marker CD20 is more often positive in PCL [17] (Fig. 1).

Three topics call for further discussion: (1) the need to examine for clonality of plasma cells in PCL, (2) the necessity of requiring a high percentage of circulating plasma cells as well as a total plasma cell count, and (3) defining the correct threshold for circulating plasma cells.

### Clonality of Plasma Cells in Plasma Cell Leukemia

The original Kyle’s criteria never described a need to investigate plasma cell clonality when defining PCL [10]. At that time, this made sense. Cell sorting, flow cytometry, and immunohistochemistry were in their absolute nascence with the first primitive electric cell sorting device being reported in 1965 [18]. Yet, we know that high amounts of circulating plasma cells are not limited to PCL, but are also observed in severe infections, mononucleosis, and serum sickness [14, 15]. Furthermore, there have been case reports of benign polyclonal plasmacytosis in other diseases, such as renal amyloidosis [16]. PCL patients also quite often present with concomitant severe infections, which may increase the number of polyclonal plasma cells [14, 15, 19•, 20•, 21•, 22•, 23•, 24•]. Therefore, clonality assessment should be included in the diagnostic work-up of plasma cell cytosis [25]. This is most efficiently done by flow cytometry.

### Defining the Requirements for Plasma Cell Leukemia Diagnosis

It has been disputed if at least 20% circulating plasma cells AND a total plasma cell count of at least 2 × 10^9^/l are required, as in the original Kyle definition, [10] or if one of these is sufficient [25]. As of now, the International Myeloma Working Group (IMWG) [25] and WHO [17] both suggest that either one of the two criteria is sufficient for the PCL diagnosis. This has, to some extent, changed the field, and it may be difficult to compare some newer studies using the new criteria to older studies that used the old, strict definitions.

### Finding a Prognostic Threshold for Circulating Plasma Cells

Identifying the most relevant threshold for circulating plasma cells to define PCL remains an interesting topic in PCL. The traditional cutoff set by Kyle et al. [10] was arbitrary, and several studies have questioned this cutoff [11, 12•,^13^•]. In 2013, it was suggested by the IMWG that thresholds for diagnosis should be reevaluated [25]. In 2017, Granell and co-workers [12•] explored 482 MM and 5 PCL patients who were classified according to the amount of circulating plasma cells (CPC) into subgroups with 0%, 1–4%, 5–20%, and PCL (≥ 20%) [12•]. It was found that 12 patients with 5–20% circulating plasma cells had a median overall survival of only 6 months. This was even lower than what was observed in the PCL patients, but the findings should of course be interpreted with caution, considering the small number of patients in the subgroups. Based on their data, Granell et al. suggested a cutoff of 5% for the definition of PCL [12•]. Likewise, Gang et al. in 2014 compared 767 patients with MM to 33 patients with PCL [26•]. MM patients with circulating plasma cells as low as 2% in blood smears had progression free and overall survival rates comparable to those of the PCL patients [26•]. In a study from The Mayo Clinic 2014, using flow cytometry, it was found that as few as 400 clonal plasma cells per 150,000 events, corresponding to 0.26% circulating plasma cells, were highly significant for reduced overall survival [27].

Also, in other settings, the prognostic impact of circulating plasma cells has been highlighted. At the time of ASCT, circulating plasma cells are indicative of short progression free survival [28]. In smoldering MM, it has been shown that circulating plasma cells identify patients with high risk of early progression to MM [13•]. Thus, several studies have shown that circulating plasma cells in MM at a threshold lower than 20% or 2 × 10^9^ identify patients with a severe and PCL-like prognosis and thereby challenge the current definition of PCL. However, a new optimal threshold has still to be determined and defined in consensus.

## Clinical Presentation

The clinical presentation of PCL differs from that of MM in several ways, and in Table 1, we have summarized the major differences. The median age at diagnosis is about 61 years, which is about 10 years less than in MM. Light chain only PCL is more common than in MM, being the second most common subtype after the IgG subtype. Also, the non-secretory subtype is more commonly observed in PLC [1, 13, 20–24]. Most PCL patients present with high tumor burden, e.g., 67% have International Staging System (ISS) stage 3 at diagnosis. Elevated lactate dehydrogenase (LDH) is common; most present with cytopenia and extramedullary involvement of the liver, spleen, and other organs besides the blood is common [1, 13•, 20•, 21•,^22^•, 23•, 24•]. Opposite, osteolyses are seen less frequently in PCL [1, 13•, 20•, 21•, 22•, 23•, 24•]. Disease presentation of PCL has been compared to that of MM in Table 1 [29–34]. Considering the very high rate of extramedullary disease, IMWG has suggested that FDG-PET/CT should be considered in diagnosis, evaluation, and monitoring of PCL [25].

**Table 1:** Clinical characteristics and cytogenetics in primary plasma cell leukemia and multiple myeloma

Clinical characteristics at diagnosis	Primary plasma cell leukemia	Multiple myeloma
Male	55%	55%
Age (median)	61.5 years	69 years
IgG	46%	58%
IgA	13%	22%
Light chain only	30%	15%
Nonsecretory	10%	4%
Other	1%	1%
Anemia, Hgb < 10 g/dL	81%	47%
Trombocytes < 130*	63%	5%
Elevated creatinine**	22%	24%
Abnormal LDH***	60%	12%
Hypercalcemia****	27%	12%
Bone disease	65%	77%
ISS I	10%	27%
ISS II	23%	39%
ISS III	67%	34%
Cytogenetic findings at diagnosis		
Translocation (11;14)	26%	21%
Translocation (4;14)	14%	14%
Translocation (14;16)	20%	4%
Deletion (17p)	40%	11%
Whole/partial deletion 13q	42%	48%
Amplification 1q	32%	40%

Data compounded from studies referenced below. *Some studies use thrombocytes < 100, **Creatinine > 2 mg/dl, ***Most studies are not defining limits of elevated LDH, ****Non ionized calcium > 2.75 mmol/l or ionized calcium > 1.45 mmol/l. References: [19•, 20•, 21•, 22•, 23•, 24•, 29, 30, 31, 32, 33, 34, 35•, 36]

## Disease Biology

The most characteristic cytogenetic findings in PCL are summarized in Table 1. Importantly, no mutations or other gene aberrations are specific for PCL compared to MM, but the relative occurrence of changes differs between PCL and MM (Table 1). Characteristic mutational patterns in PCL underline that PCL and MM are distinct entities, not only clinically but also genetically.

As in MM, translocations involving chromosome 14, t(11;14), t(14;16), and t(4;14) are common in PCL [19•, 20•, 23•, 24•, 35•, 36]. Of these t(11;14) is known to be of clinical importance in MM and other hematological diseases where it predicts sensibility to the bcl-2 inhibitor, venetoclax [37], while t(4;14) and t(14;16) are known to predict high-risk disease in MM [38].

TP53 and DIS3 mutations are more common in PCL than in MM, whereas NRAS, KRAS, and BRAF mutations are less frequently observed in PCL than in MM and sPCL [39]. A recent study using next-generation sequencing found that TP53 mutations were negatively associated with KRAS mutations and a predictor of more aggressive disease [40]. Besides being frequently mutated, TP53 located on 17p13 is also often deleted [12•, 19•, 20•, 22•]. Other deletions are also common in PCL, including 1p, 6q, 8p, 13q, 14q, and 16q [36]. MYC rearrangements have earlier been found to be commonly upregulated in PCL [41], which has also been found by Royer et al. [19•].

Very heterogeneous mutations and complex genotypes were found in a study employing whole-genome sequencing and gene expression analysis in 12 PCL cases [39]. The authors reported that the mutation patterns were more complex compared to what is observed in MM [39]. This finding has been confirmed by other groups [23•, 24•, 35•].

Data from RNA [42] and proteome studies [43] have been presented recently with focus on the transition from MM to sPCL. Ronchetti et al. studied RNA expression (especially long non-coding RNA) in MGUS, smoldering MM, MM, PCL, and sPCL [42]. Interestingly, they found that a number of long non-coding RNA’s (lncRNA) were progressively deregulated as the dyscrasia entered a more severe stage, suggesting a possible role in the progression of dyscrasia. This indicates that although lncRNAs are, by nature, non-coding, they might have regulatory roles, though it is also possible that the finding is a mere byproduct of progression [42]. In the study by Zatula et al. [43], changes in the proteome during the transformation from MM to sPCL were reported.

## Treatment

It has been reported in several studies that a significant number of patients with PCL die within few months after diagnosis [19•, 20•,^21^•, 22•, 23•, 24•]. Due to the aggressive behavior of PCL, treatment should start as soon as possible. Treatment of PCL with traditional cytostatic chemotherapy has shown poor results. However, following the introduction of ASCT, the proteasome inhibitor bortezomib and the immuno-regulatory drugs thalidomide and lenalidomide, the prognosis has, to some extent, improved.

In a major retrospective registry study in 2014 from the SEER database, Gonsalves et al. investigated the overall survival in a total of 445 PCL cases through four time periods; 1973–1995, 1996–2000, 2001–2005, and 2006–2009 [1]. Thus, the study investigated the development in survival after the introduction of ASCT became widespread in 1995, thalidomide in 2000, and bortezomib and lenalidomide in the latter period. The OS observed was only 5, 6, 4, and 12 months in the different time periods. These data do not support improved survival with the usage of ASCT or allogenic stem cell transplantation (alloSCT) but possibly indicating effectiveness of bortzemib and lenalidomide. Improved survival with ASCT and/or alloSCT has however been shown in other studies as discussed later [20•, 21•, 22•, 23•].

Very early death is a significant problem in PCL, but in the SEER data, it was also observed that the number of patients who died less than 1 month after diagnosis has decreased from 28%, 23%, 27%, and 15%, respectively, during the time periods. Unfortunately, in 2009, PCL and MM were grouped together in the SEER database, making more recent investigation difficult [1].

Fast responses, but frequent and early relapses are hallmarks of the treatment challenges in PCL [1, 13•, 20•, 21•, 22•, 23•, 24•]. Tumor lysis syndrome at the start of treatment is not uncommon, and precautions should be made. Early effective treatment must be consolidated and maintained. Different strategies for this have been evaluated as discussed in the following sections. Standard maintenance known from MM is not likely to be sufficient [1, 13•, 20•, 21•, 22•, 23•, 24•].

Table 2 presents a summary of the major clinical studies done on PCL.

**Table 2:** Overview of prospective and retrospective treatment studies in primary plasma cell leukemia. Studies performed and reported within the last 5 years including at least 20 patients have been included

Prospective studies	Number of patients	Treatment regimens	Data collection	Median PFS (months)	Median OS (months)	Major findings
Royer et al 2016 [19•]*,* French IFM group	40	PAD/VCD induction followed by HDM/ASCT and either RIC -ALLO or second HDM/ASCTand bortezomib/lenalidomide maintenance	2010–2013	15.1	36.7	- AlloSCT not shown to improve survival compared to ASCT - Bortezomib + dexamethasone plus cyclophosphamide or doxorubicin followed by ASCT improves PFS
Gang et al 2015 [26•], China	33	Bortezomib based arm or thalidomide based arm ± ASCT; arms not compared*	2004–2012	12	15	MM with circulating plasma cells has poor survival even comparable to PCL
Musto et al 2014 [22•] Italian GIMEMA Group	23	Lenalidomide and dexamethasone induction followed by single or double ASCT	2009–2011	14	28	Lenalidomide and low-dose dexamethasone induce high overall response rate but high early relapse rate. Consolidation with SCT increase PFS and OS
Retrospective studies						
Jurczyszyn et al 2018 [24•], international multicenter study	117	76% received novel treatment; 64% had upfront ASCT	2006–2016	Unknown	23	- Novel treatments induce good initial responses - ASCT predicts better OS
Katodritou et al 2018 [21•], Greek MM Study Group	50	80% received novel treatments, mostly bortezomib-based regimens; 40% underwent ASCT	2000–2016	12	18	- Bortezomib-based therapy + ASCT predicts better OS - Bortezomib decrease early mortality
Ganzela et al 2018 [20•], Israeli MM Study Group	39	Bortezomib 77%, ImiDs 67%, cyclophosmide 67%, antracyclin 26%, mephalan 13%. SCT 49% 1/3 of these allogenic	2002–2016	Unknown	15	- Bortezomib decrease early mortality - SCT predicts better survival
Jung et al 2017 [23•], Korean MM Working Party	69	25% novel agents + ASCT, 12% conventional chemotherapy + ASCT, 36% novel therapies only, 27% conventional chemotherapy only	1998–2015	12.2	16.1	- Treatment with novel agents predicts survival - Early mortality lower in patients treated with novel therapy
Iriuchishima et al 2015 [35•], Japanese MM Society	38	64% treated with vincristine, doxorubicin and dexamethasone, 34% treated with novel treatments ± ASCT/alloSCT	2001–2012	Unknown	34.2	- Novel agents significantly increase survival

*OS*, overall survival; *PFS*, progression free survival; *PAD*, bortezomib + adriamycin + dexamethasone; *VCD*, bortezomib + cyclophosphamide + dexamethasone; *HDM*, high-dose melphalan; *RIC-ALLO*, reduced-intensity conditioning allograft; *SCT*, stem cell transplantation; *ASCT*, autologous stem cell transplantation; *AlloSCT*, allogenic stem cell transplantation

*This study does not compare treatment modalities and instead focuses on prognostic value of levels circulating plasma cells in myeloma

### Bortezomib

In MM, proteasome inhibition with bortezomib has shown the ability to (partly) overcome the prognostic adverse impact of high-risk cytogenetic aberrations such as t(4;14), t(14;16), t(14;20), del(1p), and del(17p) [44–46]. Adverse cytogenetic findings are common in PCL, and bortezomib might be particularly well suited to include in the treatment. The first major study showing promising results was conducted by the GIMEMA group in 2012 [47]. In 2016, a French prospective phase 2 study tested the efficacy of bortezomib in combination with dexamethasone, and either doxyrubicin or cyclophosphamide followed by high-dose melphalan and ASCT. This study showed a high overall response rate (69%) and OS of 36.3 months [19•]. Most retrospective studies have supported an important role of bortezomib in PCL treatment [21•, 23•, 24•]; only an Israeli study did not report improved survival after treatment with bortezomib or carfilzomib [20•]. The results of these studies are summarized in Table 2.

### Thalidomide and Lenalidomide

Thalidomide and lenalidomide are IMIDs (immune modulatory drugs) that for several years have been the backbone in MM treatment. The first prospective study of lenalidomide in PCL was reported in 2014 by Musto et al. and showed an overall high response rate of lenalidomide in combination with low-dose dexamethasone [22•]. In other studies, thalidomide and lenalidomide have been found to increase survival in combination with or comparable to bortezomib [23•, 35•]. A particular role for lenalidomide and other IMIDs could be to maintain achieved response after initial treatment, and for enhancing graft-versus-leukemia effect after allo-SCT [20•].

### ASCT

Two prospective phase 2 studies have indicated that ASCT is able to prolong PFS and OS in PCL [19•, 22•]. Also, several recently published retrospective studies including population-based data reported improved PFS and OS in ASCT-treated patients [20•, 21•, 23•]. The findings in these studies are summarized in Table 2.

### AlloSCT

AlloSCT, unlike ASCT, employs the graft versus leukemia effect and is used to obtain cure in some hematological diseases. In a retrospective study from the Center for International Blood and Marrow Transplant Research, it was reported that both ASCT and alloSCT seem to improve survival; however, ASCT showed better OS rates [48]. In the prospective French trial, it was also observed that ASCT patients in fact had better PFS and OS compared to allo-treated patients [19•, 49].

### AlloSCT Compared to ASCT

So far, published data seem to favor ASCT compared to alloSCT. However, no prospective studies have made a direct comparison. AlloSCT has mostly been used in combination with ASCT, using ASCT to deepen response before alloSCT administered with reduced intensity conditioning [19•, 20•, 22•]. The poorer reported results with alloSCT could partly be caused by selection bias where patients with particularly aggressive disease behavior have been allotransplanted. Another reason for poorer outcome after alloSCT is high treatment-related mortality. Historically, this has been high, but within the last decennium, it has decreased. The potential role of alloSCT has not been finally settled. Graft-versus-tumor effect after donor lymphocyte infusion has been documented in MM [50, 51], but could be less in PCL. PostSCT maintenance could improve disease control until the GVL effect is mature, and moreover, lenalidomide, other IMId, or immunotherapy could enhance the GVL effect [52]. Ongoing trials, including the European primary PCL study (EudraCT number 2013-005157-75) will contribute to clarify the role of alloSCT in PCL.

### Maintenance Treatment

Early progression of PCL after achieved remission is the norm even after deep responses. Therefore, maintenance therapy is needed after ASCT and after end of induction in patients not eligible for ASCT. In the prospective study by Musto et al., maintenance treatment was given as lenalidomide 10 mg/day 1–21 of 28-day cycles. Still, 50% of patients relapsed within 12 months after the start of maintenance [22•]. In the French prospective study, lenalidomide, bortezomib, and dexamethasone were given as maintenance. Only one of seven patients relapsed during the study period; three had to stop maintenance therapy due to prolonged cytopenia [19•]. In a Japanese retrospective study, it was reported that patients who received maintenance with bortezomib, thalidomide, or lenalidomide tended to have longer OS, 4.5 versus 2.9 years, though the number of patients was too small for this to be significant [35•]. The low numbers of included patients in studies make definite conclusions difficult. However, the current best strategy for maintaining PCL patients in remission seems to be giving a combination of bortezomib and lenalidomide.

### Treatment of sPCL

For sPCL, studies are extremely limited, and patients are often heavily pretreated. A recent study indicated improved prognosis by treatment with bortezomib-containing regimens. The study reported the most important factor to be high quality first response to treatment [53]. A recent, small study further investigated treatment of sPCL with bortezomib and lenalidomide-containing regimen achieving PFS of more than 27 months in 2/9 pts. [54]. As in MM, treatments with thalidomide and lenalidomide are likely to have some effect on sPCL, but patients will often already have received these treatments [54]. ASCT has been used for sPCL. The survival was still poor, but a few patients achieved remission for more than 1 year [54].

### New and Upcoming Treatments and Studies

Venetoclax is a BCL-2 inhibitor that has demonstrated remarkable efficacy in MM, CLL, and other hematological diseases harboring the (11;14) translocation [37]. As noted earlier, this particular translocation is common in PCL. In a recent case report, venetoclax was used in combination with daratumumab, dexamethasone, and bortezomib in a t(11;14) refractory PCL patient resulting in a rapid and deep response already after the first treatment cycle [55].

Pomalidomide is a third-generation IMID that has shown good response and survival benefit in refractory MM [56, 57]. In a case report, a PCL patient with CNS relapse after allogenic SCT was successfully treated with cerebral radiation and intrathecal chemotherapy followed by pomalidomide and dexamethasone maintenance. At the time of reporting, the patient was still in remission after 18 months of follow-up [58]. In another case report, a patient with sPCL achieved normalization of hematological values and significant decrease in M-component after 4-month treatment combining low-dose dexamethasone and pomalidomide [59].

Ixazomib is a second-generation PI used in combination with lenalidomide and dexamethasone in relapsed or refractory MM [60, 61]. Ixazomib is currently being investigated in a phase 1b study as maintenance treatment after alloSCT in relapsed high-risk MM included patients with sPCL and PCL (NCT02504359) [62]. An ongoing phase II study from the Mayo Clinic investigates the efficacy of combining ixazomib, pomalidomide, and dexamethasone for sPCL or previously treated MM (NCT02547662) [63].

Carfilzomib, another second-generation PI, combined with lenalidomide and dexamethasone is currently being tested as induction treatment of PCL in a European multi-center study (EudraCT number 2013-005157-75). Responding transplant eligible patients are subsequently treated with ASCT followed by allo-SCT, and hereafter maintained with low-dose lenalidomide to increase GVL effect.

Daratumumab is an anti-CD38 antibody which, in several studies, has shown impressive efficacy in relapsed, refractory MM. Daratumumab and other anti-CD38 antibodies will for sure be of high interest to study in PCL and sPCL (NCT03591744) [64].

The use of anti-CD45 antibodies is currently being investigated for high-risk myeloma, [65], and other antibodies-targeting CD75s are currently being investigated for their ability to bind MM and PCL cells [66].

BRAF/MEK inhibitors are newly developed compounds that are used successfully for targeted treatment of malignant melanoma [67] and MM [68]. BRAF pathway mutations are seen in about 5–6% of MM patients, whereas BRAF pathway mutations are less frequently observed in PCL compared to sPCL and MM. The treatment principle of combining BRAF and MEK inhibitors will be an interesting option in the treatment of BRAF pathway-mutated PCL or sPCL patients [68].

CAR-T therapy is an exciting new technique using genetically engineered autologous T cells that are programmed to bind specific antigens on target cells. Encouraging results have been found in lymphomas and leukemia and also in MM [69]. Data in the PCL setting is pending.

Peptide vaccination studies have so far not fulfilled their promises in MM. However, studies are ongoing, also including PCL patients [70].

## Conclusion

In conclusion, the diagnostic criteria of PCL are under discussion in the international myeloma community. Both primary and particularly secondary PCL are very aggressive diseases with adverse prognoses. Secondary PCL is often treatment resistant, whereas early and even deep responses are common in primary PCL, but early relapses and development of resistance are typical. Treatment should start promptly with an effective proteasome inhibitor-containing regimen and followed by high-dose chemotherapy and ASCT in eligible patients. A strategy for further semi-intensive consolidation and continued maintenance should always be considered. AlloSCT has so far shown less encouraging results, but might be considered in younger patients. A number of new treatment modalities are currently being investigated and will hopefully be able to improve the prognosis in this devastating disease.

## Abbreviations

PCL: Primary plasma cell leukemia
MM: Multiple myeloma
sPCL: Secondary plasma cell leukemia
PI: Proteasome inhibitor
OS: Overall survival
PFS: Progression-free survival
IMID: Immunomodulatory drugs
SCT: Stem cell transplantation
ASCT: Autologous stem cell transplantation
alloSCT: Allogeneic stem cell transplantation
